# An Assessment of Health Related Quality of Life in a Male Prison Population in Greece Associations with Health Related Characteristics and Characteristics of Detention

**DOI:** 10.1155/2014/274804

**Published:** 2014-07-02

**Authors:** Constantinos Togas, Maria Raikou, Dimitris Niakas

**Affiliations:** ^1^Faculty of Social Sciences, Hellenic Open University, 26335 Patras, Greece; ^2^LSE Health, London School of Economics and Political Science, London WC2A 2AE, UK

## Abstract

*Background*. Prisoners constitute a group with increased health and social care needs. Although implementing policies that aim at improving outcomes within this population should be a priority area, studies that attempt to assess health outcomes and health related quality of life (HRQoL) in this population are limited. *Aim*. To assess HRQoL in a prison population in Greece and to explore the relationship between HRQoL and a set of individual sociodemographic and health related characteristics and characteristics of detention. *Methods*. A cross-sectional study involving 100 male prisoners was conducted in the prison of Corinth in Greece. HRQoL was assessed through the use of the SF-36 and the EQ-5D. *Results*. The mean physical and mental summary scores of the SF-36 were 55.33 and 46.82, respectively. The EQ-VAS mean score was 76.41%, while the EQ-5D index was 0.72. Multivariate analysis identified a statistical relationship between HRQoL and the conditions of detention, controlling for the effect of sociodemographic characteristics, morbidity, and mental problems. The use of narcotics in particular is significantly associated with lower HRQoL.* Conclusions*. Implementation of policies that aim at preventing the use of narcotics within the prison environment is expected to contribute to improved HRQoL in this population.

## 1. Introduction

According to the latest World Health Organization (WHO) publication on imprisonment, the estimated imprisoned population across the WHO European Region on any given day in 2012 was 2 million people [1]. Taking into account the rate of turnover among this population, it is estimated that a total of 6 million people are imprisoned at some point during a given year. The average prison population rate across the Region is estimated to be 150 prisoners per 100,000 inhabitants (0.15%), ranging from less than 10 prisoners per 100,000 inhabitants (0.01%) to almost 600 prisoners per 100,000 inhabitants (0.6%). Furthermore, in the majority of the WHO European Member States, the prison population has been shown to be increasing over the last decade. The majority of the prison population comes from poor and deprived and vulnerable social groups and approximately 21% are foreign nationals. As a result, they are at a higher risk of communicable and noncommunicable diseases compared with people in the general population [1].

Studies have consistently shown that the rates of Human Immunodeficiency Virus (HIV) infection, hepatitis B and C, and tuberculosis (TB) among prisoners in all countries are significantly higher than those in the general population [1]. The rate of TB in particular has been reported to be 84 times higher among prisoners in the WHO European Region in 2002 than in the general population. More importantly, high transmission rates in prisons are clearly observed for TB and HIV and they have been deemed to be associated with the concentration of high risk subpopulations but also with the lack of effective control measures. Furthermore, some health conditions have been observed to be more prevalent among prisoners than in the general population. Thus, prisoners are at higher risk for cardiovascular disease and some types of cancer and for developing mental health problems and disorders. Suicide rates are also nonnegligible among prisoners in the Region with an average of 6 per 10,000 prisoners ranging from 0 (0%) to almost 30 (0.3%) [1].

These health risks tend to become even higher and more difficult to contain as prisons themselves are settings carrying high risks of disease. First, because there is continuous contact and exchange with communities outside of prison, controlling the transmission and prevalence of communicable diseases such as tuberculosis or HIV among inmates becomes very complex. Second, it is very common that the conditions of imprisonment are themselves unhealthy being characterised by lack of space, sanitary facilities, light, and fresh air. In addition, many prisons in the WHO European Region lack qualified staff especially health and social care staff. As a result, the health and social care services provided to prison population are typically inferior to those provided to the general population [1].

Mental health problems can be present at the time of imprisonment or develop during the period of imprisonment and become aggravated due to the prison environment and the specific conditions of detention [2, 3], leading to high levels of morbidity due to mental health conditions [4]. The most prevalent habits among the prison population are smoking, the use of drugs and narcotics, and the consumption of alcohol [5, 6], all of which are well known to be negatively associated with health status.

Overall, the prison population is a vulnerable population with increased health needs compared to the general population. These needs are very inadequately met at present leading to a prison environment that is characterised by high transmission rates of communicable disease, increased risks of mental disease and specific health conditions, stressful and violent conditions of detention, and poor if not completely lacking measures to control detrimental health habits [7, 8]. As a result, prisoners' health and health related quality of life (HRQoL) can be significantly affected by the prison environment [9].

Against this background, the aim of this study was to assess HRQoL in a prison population in Greece and to explore the relationship between HRQoL and a set of individual sociodemographic and health related characteristics and characteristics of detention.

## 2. Methods

### 2.1. Study Design and Questionnaire

A cross-sectional study was conducted from February 2013 to May 2013 in the prison of Corinth in the region of the Peloponnese in Greece. Ethical approval for the study was obtained from the Ministry of Justice. All prisoners serving a sentence or awaiting sentence were eligible to participate in the study. Those who were transfers from other prisons awaiting trial and would only stay for a short period in the prison of Corinth were excluded from the study as they were deemed to be nonrepresentative of the prison population due to their transient status. In addition, as the questionnaires used for the purpose of this study have been validated and extensively used in the English and the Greek language, non-English and non-Greek speaking inmates were excluded. This resulted in a total of 30 individuals being excluded, leaving 100 prisoners in the study sample who after having received information about the study objective and design agreed to participate in the study. Each participant was then administered and completed the study questionnaire, in the presence of the social worker of the prison who was also responsible for the collection and collation of completed questionnaires.

The first part of the questionnaire included a section on sociodemographic characteristics, a section relating to the specific sentence and conditions of detention and a section on health related characteristics and conditions.

The second part comprised of the SF-36 and the EQ-5D HRQoL instruments. The SF-36 is a 36-item, short-form, generic health survey. It yields an 8-scale profile of functional health and well-being scores (scales), namely, physical functioning (PF) and social functioning (SF), physical role (RP) and emotional role (RE), bodily pain (BP), general health (GH), mental health (MH) and vitality (VT), as well as two psychometrically based summary measures, those of physical health (PCS) and mental health (MCS) [10]. The SF-36 shows high reliability and convergent and discriminant validity [11–13] and it has been widely used and validated for the Greek population [13, 14].

The EQ-5D is also a generic instrument that has been validated for the Greek population [15, 16] and comprises two parts. The first part is a descriptive system of five dimensions of health, namely, mobility, self-care, usual activities, pain/discomfort, and anxiety/depression. The second part constitutes a visual analogue scale (VAS), the EQ-VAS, that asks the respondents to self-rate themselves on a thermometer-like grading system in order to capture variations in health states. Furthermore, EQ-5D health state valuations may be converted into a single summary index by applying an algorithm that attaches values to each of the levels in each dimension. Value sets have been derived for the EQ-5D scores in several countries using either the standard gamble or the time trade-off (TTO) valuation technique.

Comparison of the two methods suggests that the TTO performs slightly better in terms of the internal consistency of the answers given by respondents, the sensitivity of valuations to parameters known to influence them, and the reliability of the responses when the valuation task was repeated by the same respondents some weeks later [17]. Thus, analysis was based on valuations generated by the TTO method which asks respondents to sacrifice quantity of life (i.e. life expectancy) for quality of life [17]. In this study, due to the lack of an available value set for the Greek population, an EQ-5D index was derived based on the TTO valuation technique applied to the United Kingdom population (York A1 Tariff) [17].

### 2.2. Statistical Analysis

A descriptive analysis of the distribution of the variables of interest and an analysis of correlation between the variables of interest were undertaken. Reliability was tested via Cronbach's coefficient alpha (*α*). Linear regression analysis was used to study the relationship between HRQoL and the following set of independent variables: age, ethnicity, marital status, level of education, work while being in prison, having visits while being in prison, previous imprisonment, release on temporary licence (ROTL), receiving psychiatric medication, total number of health conditions (diagnoses) as a measure of morbidity, smoking status, and years of narcotics use. The analysis was conducted using Stata 12.0.

## 3. Results

Table 1 summarises the major sociodemographic characteristics, health related characteristics, and characteristics related to imprisonment and conditions of detention of the sample population. In total 100 prisoners, all men (the prison in Corinth is a male prison), were enrolled in the study and all returned completed questionnaires (response rate 100%). There were no missing data.

### 3.1. Sociodemographic Characteristics

The mean age of the sample was 34.07 years (SD 9.39 years) with the majority of the participants being between 21 and 40 years old (no inmate was below 21 years of age as the prison of Corinth is an adult prison and according to the Greek prison system anyone younger than 21 years will be detained in a juvenile detention centre). Most of the participants (63%) were Greek, 10% Albanian, 5% Romanian, 4% Pakistani, 3% Afghan, 3% Iraqi, 2% Georgian, 2% Nigerian, 2% Algerian, 1% Bulgarian, 1% Turkish, 1% Syrian, 1% Palestinian, 1% Guinean, and 1% Moroccan. Most inmates were not married, had received only primary education, and are thus considered to be of a low educational achievement (The Greek education system is mainly divided into three levels, primary, secondary, and tertiary. Primary education is divided into kindergarten and primary school lasting one and six years, respectively. Secondary education comprises a three-year middle high school and a three-year academically oriented high school after which students can attend university (tertiary education)) and consistent with a low educational achievement, their occupation prior to their imprisonment was manual (manual occupation identifies farmers, unskilled labourers, and skilled manual employees. Nonmanual occupation identifies skilled nonmanual self-employed professionals and employees performing managerial or administrative work in the public or the private sector).

### 3.2. Health Related Characteristics

The mean body mass index (BMI) was 25.68 kg/m^2^ (SD 3.69 kg/m^2^) with 34% of the participants being overweight and 13% being obese. A total of 77 health related conditions were recorded among the prisoners with the most frequently reported being mental disorders accounting for about 25% of the reported conditions, hepatitis C also accounting for about 25% of the reported conditions, and hepatitis B accounting for 10% of the reported conditions. Half of the prisoners (51%) did not report a health related condition, 26% reported one condition, 19% two, 3% three, and 1% four health related conditions. A total of 27% were taking medications of whom the majority (approximately 60%) were receiving psychiatric drugs alone, 30% were administered medication for health conditions other than mental problems, and 11% were receiving medication for both nonmental and mental health problems.

### 3.3. Characteristics Relating to Imprisonment and Conditions of Detention

At the time of the study, the majority of the prisoners (36%) were sentenced to an imprisonment of 5 to 10 years, 27% faced a sentence longer than 10 years, 6% less than 5 years, and 4% lifetime. The remaining 27% were (in custody) awaiting sentence.

Half of the prisoners (49%) had been imprisoned in the past, 42% had been receiving visitors while being in prison, and 26% worked while being in prison. A small proportion (7%) was allowed to leave the prison on temporary license regularly (i.e., for day release, overnight release, or some other situation; they were then obliged to return to prison) and 6% were awaiting immediate release. At the time of the study, this sample population of prisoners had been in prison on average for 26 months (SD 25.74 months) and only 9% had served 5 years or more.

The majority of the inmates were smokers (88%) and did not want to quit (68%). Almost half of the prisoners (47%) were users of narcotics and had been taking them regularly for an average of 13 years (mean: 13.34 years, SD 6.87 years). The most frequently used substance was cannabis (39 out of 47, that is, 83%). This was followed by opiates-opioids (33 out of 47, that is, 70%), stimulants of the central nervous system (28 out of 47, that is, 60%), sedatives of the central nervous system (21 out of 47, that is, 45%), and hallucinogenics (8 out of 47, that is, 17%). The majority were frequent users of multiple substances.

### 3.4. Quality of Life

#### 3.4.1. SF-36 and EQ-5D Valuations

Table 2 presents the valuations for each scale of the SF-36 and the scores of the summary components. It also presents the valuations based on the EQ-5D for each dimension and the EQ-VAS and the EQ-5D index scores. Overall, it can be seen that the prisoners of the sample report relatively high values in all scales of the SF-36 instrument with the exception of the mental health scale (MH). The highest mean score was reported in the physical functioning (PF) scale (mean 90.45, SD 19.3) and the lowest mean score was reported in the mental health (MH) scale (mean 60.76, SD 23.5). For the remaining scales of the SF-36, the mean scores vary between 76.19 (SD 19.2) for GH and 88.94 (SD 21.6) for BP. The summary component measures mean scores were estimated to be 55.33 (SD 8.68) for PCS and 46.82 (SD 11.54) for MCS.

The EQ-5D index mean summary score was 0.724 (SD 0.281, min: −0.181, max: 1) using the York A1 Tariff TTO valuation technique. The mean VAS score was 76.41 (SD 20.35, min: 10, max: 100). Among the different EQ-5D dimensions, the majority of the prisoners had no problems with mobility (87%), self-care (94%), usual activities (91%), or pain/discomfort (77%). By contrast, for the dimension of anxiety/depression 72% of the respondents reported having some/extreme problems.

The most frequently reported health state (36%) was having no problems in the dimensions of mobility, self-care, usual activities, or pain/discomfort and some problems in the dimension of anxiety/depression. It is also worthwhile mentioning that a substantial proportion (23%) of the respondents reported considering themselves in a state of full health (i.e., reported having no problems in all dimensions of the EQ-5D).

#### 3.4.2. Assessment of Consistency

Internal consistency of the instruments was assessed by Cronbach's coefficient *α*. This was estimated to be 0.895 for the SF-36 instrument well exceeding the level of acceptance of 0.700 for group level comparisons. Cronbach's *α* coefficient for the EQ-5D was 0.633 also approaching the level of acceptance.

Strong correlation was detected between the EQ-VAS and the EQ-5D York A1 Tariff (*r* = 0.7453; *P* < 0.001) indices. In general, strong and highly statistically significant correlations were detected between the scales and the dimensions/indices of the two instruments. Specifically, dimensions and scales that assess the same aspect of quality of life demonstrated stronger negative correlations (e.g., mobility and PF, *r* = −0.7463, *P* < 0.001) in contrast to less relevant ones (e.g., mobility and MH; *r* = −0.1401, *P* = 0.1646). Overall, the EQ-VAS and the EQ-5D indices revealed highly statistically significant positive correlations with all of the SF-36 scales.

Both HRQoL instruments presented satisfactory levels of acceptance, sensitivity, reliability, and validity. Internal consistency for both instruments as measured by Cronbach's coefficient *α* reached satisfactory levels and convergent validity was shown through the expected pattern of highly statistically significant correlations that were detected between the scales and the dimensions/indices of the two instruments. These results on the instruments' levels of sensitivity, reliability, validity, and internal consistency can be seen as a strength of the study's analytical methodology.

#### 3.4.3. Correlations between HRQoL and Sociodemographic, Health, and Detention Characteristics

Table 3 presents the correlation coefficients between the EQ-5D tariff index, the two summary scores of the SF-36, and a number of sociodemographic and health characteristics as well as characteristics of detention.

In general, the correlation coefficients are of the expected sign. Concentrating on the variables where the estimated correlations attain statistical significance at the 5% level the main findings follow. Age is negatively correlated with all three measures of HRQoL but the correlation is significant only with respect to the SF-36 PCS score. As expected, a negative correlation is also detected between the total number of health conditions (as a proxy for morbidity) a person is diagnosed with and all three measures of HRQoL with the correlation being significant with respect to the EQ-5D tariff and with respect to the SF-36 PCS score. Having had a previous imprisonment is also negatively correlated with all three measures but the correlation is significant only with respect to the EQ-5D tariff.

More importantly, the conditions of detention also appear to play a role in determining quality of life. Thus, working while in prison is positively correlated with all three measures although the correlation is significant only with respect to the EQ-5D tariff. Being allowed to leave the prison regularly on temporary license is also positively correlated with all three measures and the correlation is significant with respect to the EQ-5D tariff and the SF-36 MCS score. Importantly, the use of narcotics is negatively correlated with all three measures of HRQoL and the correlation is statistically significant with respect to the EQ-5D tariff and the SF-36 MCS score. Finally and related to this, the duration (years) of narcotics use is negatively correlated with all three HRQoL scores and this correlation is statistically significant with respect to all three measures of HRQoL.

#### 3.4.4. Regression Analysis

Following the above findings, regression analysis was used to study the statistical relationship between HRQoL and the conditions of detention while accounting for the effect of sociodemographic and health related individual characteristics. Multivariate linear regression models were estimated one for each measure of HRQoL. The results of the linear regression analysis for all three measures of HRQoL are presented in Table 4. Note that HRQoL scores are expressed on a scale from 0 to 1 and on a scale from 0 to 100 using the EQ-5D and the SF-36 instruments, respectively.

Concentrating first on the coefficients which attain statistical significance at the 5% level, the results show that prisoners who are allowed to leave the prison on temporary license regularly report a higher EQ-5D score compared to those who are not and that the duration of the use of narcotics is associated with a lower EQ-5D score. At a 10% level of significance, the results show statistical associations between HRQoL and the level of education, with those having higher education reporting a lower EQ-5D score, whether the prisoners work in prison or not, with those working while in prison reporting a higher EQ-5D score than those who do not, and with the degree of morbidity as proxied by the number of diagnosed health conditions, with higher morbidity being associated with a lower EQ-5D score.

The results of the linear regression on the physical summary score of the SF-36 show that the use of psychiatric medications is statistically significantly associated with better reported physical health (*P* < 0.05), and as was the case with the EQ-5D score, higher morbidity and longer use of narcotics are significantly associated with lower reported physical health (*P* < 0.01). In addition, as expected, older individuals report a lower physical health score (*P* < 0.05).

The results of the regression on the summary mental score of the SF-36 do not present a clear picture of self-reported mental health as none of the estimated coefficients are statistically significant aside from the coefficient on release on temporary licence (ROTL) which is of the expected sign and is significant at the 10% level, indicating that allowing prisoners to leave the prison regularly on temporary license is associated with better reported mental health. Moreover, as was the case with the two previous measures of HRQoL, the years of use of narcotics appear to be associated with worse mental health; however, the estimate is not statistically significant. The fact that the results here did not attain statistical significance might be explained by the small sample size or by the possible stigma associated with mental health reporting.

Based on the results of the regression analysis on all three indices of HRQoL, the main finding is that there appears to be a statistical relationship between HRQoL as measured by the above instruments and the use of narcotics while controlling for individual sociodemographic and health related characteristics. The use of narcotics is associated with reduced levels of HRQoL among this group of prisoners and the association becomes stronger as the duration of use increases.

## 4. Discussion

This study attempts to assess HRQoL in a prison population in Greece and to explore the relationship between HRQoL and a set of individual sociodemographic and health related characteristics and characteristics of detention. Two generic instruments, the SF-36 and the EQ-5D, were used to evaluate HRQoL in the population detained in the prison of Corinth in the Region of the Peloponnese, over the period of February 2013 to May 2013.

Consistent with previously reported findings in similar prison populations [1–8], the most frequently recorded health related conditions were mental disorders and hepatitis C followed by hepatitis B. A significant proportion of the prisoners were receiving medications of whom the majority were receiving psychiatric drugs for mental health problems. Moreover, the majority of the inmates were users of tobacco and a substantial proportion of them were users of narcotics and had been taking them regularly over a long period. The most frequently used narcotic substances were cannabis, opiates-opioids, stimulants of the central nervous system, sedatives of the central nervous system, and hallucinogenics while the majority of the prisoners were frequent users of multiple substances.

Overall, the sample of prisoners reported relatively high values in all scales of the SF-36 instrument with the highest mean score being reported in the physical functioning (PF) scale with the exception of the mental health scale (MH) where the lowest mean score was reported. Similar results were obtained from the EQ-5D instrument on both the EQ-5D index and the VAS scores. Among the different EQ-5D dimensions, the majority of the prisoners had no problems with mobility, self-care, usual activities, or pain/discomfort. By contrast, for the dimension of anxiety/depression, the majority of the respondents reported having some/extreme problems. The results derived from the use of both instruments, therefore, are consistent and they show that the study population reports relatively high levels of physical health and low levels of mental health.

Consistent with the findings reported in other studies on similar prison populations [1–9], the investigation of the relationship between HRQoL and health related characteristics identified a negative correlation between age and HRQoL and between morbidity (as proxied by the total number of health conditions a person is diagnosed with) and HRQoL. Having had a previous imprisonment appears also to be negatively correlated with HRQoL. More importantly, the conditions of detention appear to be significantly associated with HRQoL. Thus, working while being in prison and being allowed to leave the prison regularly on temporary license are both associated with better HRQoL. These findings indicate that such initiatives should be pursued as explicit policy objectives.

This study also shows that the use of narcotics is negatively associated with HRQoL and this becomes more significant as the duration of narcotics use increases. This finding holds controlling for sociodemographic and health related characteristics such as age, level of education, morbidity, and the use of psychiatric medication. If prison authorities are concerned with the prison population HRQoL, effort should be made to educate and promote policies which reduce narcotic use in this population. Improved health of the prison population will have a positive impact in the long-term not only on the public health of a deprived and vulnerable population, but also on the integration of prisoners into society on release with lower rates of reoffending and reconviction and, therefore, greater public protection.

The main limitation of this study is that it is a single-center study in a small regional prison of Greece with a relatively small sample size which consists solely of male prisoners of a relatively young age and did not include any non-English and non-Greek speaking inmates. These characteristics may restrict the generalisability of the study's results. Moreover, the questionnaires were completed in the presence of the social worker of the prison and it is possible that some prisoners may have misreported their physical and mental health state in an attempt to avoid being stigmatised or to avoid the risk of being deprived of certain privileges (e.g., being allowed to work in prison, or being allowed to leave the prison on temporary licence). Additionally, neither did this study examine the repeatability of the HRQoL evaluation due to the short study duration nor was there a matched control group drawn from the general population to allow a comparative evaluation of HRQoL in the prison population.

Moreover, self-reporting bias might be imparted, especially in the dimension of mental health. As this study showed, the results derived from the SF-36 summary mental score, although consistent with the results derived from the EQ-5D and the SF-36 summary physical score, did not attain statistical significance. This might be explained by the small sample size or it could be that there is a possible stigma associated with mental health reporting especially in a prison population where being stigmatised could have serious consequences as stated above. In a future study, special attention should be paid, therefore, to ensure mental health reporting bias is minimized.

Future research could entail the administration of the two generic instruments used here to a national representative sample of the prison population in Greece to verify the generalisability of this study's results and additionally the investigation of the change in HRQoL over time after enforcing measures to amend the conditions of detention identified to have a significant impact on HRQoL in this population.

Notwithstanding the limitations discussed above, all three indices of HRQoL estimated in this study appear to indicate the presence of a statistical relationship between HRQoL as measured by the above instruments and the use of narcotics. This is an important finding and highlights the role that policies aimed at reducing narcotics use in the prison population during time in captivity may have on their general well-being. This may even have a spillover impact on this population's longer-term narcotics use. Whilst this has not been pursued in this study, it raises the potential of policies aimed at breaking levels of narcotics use through educating the prison population on the health implications of such abuse, as part of general approaches to reducing drug abuse and related criminal activity.

## Figures and Tables

**Table 1:** Sociodemographic, health related, and imprisonment characteristics of the sample population.

Sociodemographic characteristics		Health related characteristics		Imprisonment-related characteristics	
Characteristic	Percentage	Characteristic	Percentage	Characteristic	Percentage
Gender		Obesity		Sentence	
Men	100%	Normal weight (BMI < 26)	53%	<5 years	6%
Women	0%	Overweight (BMI ≥ 26, BMI ≤ 29)	34%	5–10 years	36%
Ethnicity		Obese (BMI > 29)	13%	>10 years	27%
Greek	63%	Health conditions		Lifetime	4%
Other	37%	No health condition	51%	Awaiting sentence	27%
Age group		One health condition	26%	Previous imprisonment	
21–30 years	40%	Two health conditions	19%	Yes	49%
31–40 years	37%	>two health conditions	4%	No	51%
41–50 years	15%	Mental disorders	25%	Visits in prison	
>51 years	8%	Hepatitis C	25%	Yes	42%
Marital status		Hepatitis B	10%	No	58%
Unmarried	53%	Receiving medications	27%	Work in prison	
Married	35%	For mental problems	60%	Yes	26%
Divorced	5%	For other than mental problems	30%	No	74%
Separated	7%	For mental and nonmental problems	11%	Release on temporary license (ROTL)	
Education				Yes	7%
Not having attended school	19%			No	93%
Primary school	32%			Smoking status	
Middle high school	25%			Yes	88%
Academically oriented, high school	19%			No	12%
University	5%			Use of narcotics	
Occupation prior to imprisonment				Yes	47%
Manual	77%			No	53%
Nonmanual	16%			Cannabis	83%
Unemployed	7%			Opiates-opioids	70%
				Stimulants of the central nervous system	60%
				Sedatives of the central nervous system	45%
				Hallucinogenics	17%

**Table 2:** SF-36 and EQ-5D scores and valuations.

SF-36 scales	SF-36			
	Min	Max	Mean	SD
PF	0	100	90.45	19.26
RP	0	100	87	32.66
BP	0	100	88.94	21.59
GH	10	100	76.19	19.18
VT	0	100	76.75	28.12
SF	12.5	100	85.75	22.40
RE	0	100	82.33	32.29
MH	0	96	60.76	23.45
PCS	15.1	65.9	55.33	8.68
MCS	16.4	61.1	46.82	11.54

EQ-5D dimensions	EQ-5D			
	No problems	Some problems	Extreme problems	

Mobility	87%	13%	0%	
Self-care	94%	6%	0%	
Usual activities	91%	9%	0%	
Pain/discomfort	77%	18%	5%	
Anxiety/depression	28%	49%	23%	

	Min	Max	Mean	SD

EQ-5D index	−0.181	1	0.724	0.281
EQ-VAS (%)	10	100	76.41	20.35

*Note.* PF: physical functioning; RP: physical role; BP: bodily pain; GH: general health; VT: vitality; SF: social functioning; RE: emotional role; MH: mental health; MCS: mental component summary; PCS: physical component summary; VAS: visual analogue scale.

**Table 3:** Correlations between HRQoL and sociodemographic, health, and detention characteristics.

Variable name	EQ-5D tariff	SF-36 PCS	SF-36 MCS
	Correlation (*P* value)	Correlation (*P* value)	Correlation (*P* value)
Age	−0.0500 (0.6214)	**−0.2997** (0.0024)	−0.0263 (0.7950)
Ethnicity	0.1229 (0.2233)	0.1270 (0.2079)	0.0903 (0.3716)
Marital status	−0.0493 (0.6259)	−0.1136 (0.2606)	0.0907 (0.3695)
Educational level	−0.0409 (0.6862)	0.0706 (0.4851)	0.0985 (0.3297)
Work in prison	**0.2857** (0.0040)	0.1285 (0.2026)	0.1139 (0.2590)
Visits in prison	0.0594 (0.5569)	0.0857 (0.3964)	0.0177 (0.8613)
Immediate release	0.0395 (0.6967)	−0.0288 (0.7759)	−0.0645 (0.5236)
Sentence	0.1611 (0.1093)	0.0189 (0.8518)	0.0814 (0.4209)
Previous imprisonment	**−0.2172** (0.0300)	−0.0544 (0.5909)	−0.1458 (0.1477)
ROTL	**0.2205** (0.0275)	0.0751 (0.4577)	**0.2014** (0.0445)
Psychiatric medication	−0.1300 (0.1972)	0.0231 (0.8197)	−0.1747 (0.0821)
Number of health conditions	**−0.3146** (0.0014)	**−0.4240** (0.0000)	−0.1105 (0.2736)
Smoking	−0.0867 (0.3911)	0.0002 (0.9981)	0.0801 (0.4284)
Use of narcotics	**−0.2133** (0.0331)	−0.1480 (0.1418)	−**0.1958** (0.0509)
Years of narcotics use	**−0.3014** (0.0023)	**−0.3422** (0.0005)	**−0.2250** (0.0244)

**Table 4:** Multivariate linear regression models on HRQoL using the EQ-5D and the SF-36 instruments.

Characteristic	Outcomes								
	EQ-5D tariff∗∗			SF-36 summary physical score∗∗∗			SF-36 summary mental score∗∗∗∗		
	Coefficient	Standard error	*P* value	Coefficient	Standard error	*P* value	Coefficient	Standard error	*P* value
Age group									
Age 31–40 years versus age 21–30 years∗	0.085	0.073	0.246	1.109	2.030	0.586	−2.422	3.127	0.441
Age 41–50 years versus age 21–30 years∗	0.102	0.093	0.275	−1.908	2.596	0.464	4.581	3.999	0.255
Age > 51 years versus age 21–30 years∗	−0.058	0.116	0.619	−8.219	3.254	0.013	−9.351	5.012	0.066
Ethnicity									
Non-Greek versus Greek∗	0.046	0.075	0.544	0.289	2.094	0.891	−1.601	3.226	0.620
Marital status									
Married versus never married, divorced, separated, or widowed∗	−0.072	0.066	0.278	−2.117	1.854	0.257	1.955	2.856	0.496
Educational Level									
High school or lyceum or higher education versus no schooling or primary school∗	−0.098	0.058	0.088	−0.002	1.595	0.999	0.124	2.457	0.960
Work in prison									
Working while being in prison versus not working while being in prison∗	0.111	0.066	0.096	1.601	1.837	0.386	1.683	2.829	0.553
Visits in prison									
Having visitors while being in prison versus not having visitors while being in prison∗	0.039	0.058	0.502	−0.485	1.612	0.764	−0.646	2.483	0.795
Previous imprisonment									
Having had a previous imprisonment versus having had no previous imprisonment∗	0.003	0.073	0.966	2.539	2.044	0.218	−1.298	3.148	0.681
Release on temporary license (ROTL)									
Allowed release on temporary license versus not allowed release on temporary license∗	0.232	0.107	0.033	3.257	2.992	0.279	8.002	4.608	0.086
Psychiatric medication									
Taking psychiatric medication versus not taking psychiatric medication∗	0.056	0.081	0.497	4.751	2.275	0.040	−4.168	3.504	0.238
Health conditions									
Number of diagnosed health conditions	−0.065	0.036	0.070	−3.271	0.994	0.001	1.099	1.531	0.475
Smoking status									
Being a smoker versus not being a smoker∗	0.041	0.088	0.638	2.076	2.448	0.399	5.707	3.771	0.134
Use of narcotics									
Number of years of narcotics use	−0.009	0.004	0.027	−0.395	0.119	0.001	−0.273	0.183	0.142
Constant	0.738	0.105	0.000	57.033	2.934	0.000	44.209	4.521	0.000

*Reference category.

∗∗Number of observations = 100; Prob > *F* = 0.0075; *R*-squared = 0.2821; Adjusted *R*-square = 0.1638; Root MSE = 0.2573.

∗∗∗Number of observations = 100; Prob > *F* = 0.0000; *R*-squared = 0.4100; Adjusted *R*-square = 0.3129; Root MSE = 7.1958.

∗∗∗∗Number of observations = 100; Prob > *F* = 0.0991; *R*-squared = 0.2073; Adjusted *R*-square = 0.0768; Root MSE = 11.084.

## References

[1] WHO Good governance for prison health in the 21st century.

[2] Brown GW, Andrews B, Harris T, Adler Z, Bridge L (1986). Social support, self-esteem and depression. *Psychological Medicine*.

[3] Nurse J, Woodcock P, Ormsby J (2003). Influence of environmental factors on mental health within prisons: focus group study. *British Medical Journal*.

[4] Fazel S, Seewald K (2012). Severe mental illness in 33,588 systematic meta-regression analysis. *The British Journal of Psychiatry*.

[5] Richmond RL, Butler TG, Indig D, Wilhelm KA, Archer VA, Wodak AD (2012). The challenges of reducing tobacco use among prisoners. *Drug and Alcohol Review*.

[6] Fazel S, Bains P, Doll H (2006). Substance abuse and dependence in a systematic review. *Addiction*.

[7] Conklin TJ, Lincoln T, Tuthill RW (2000). Self-reported health and prior health behaviors of newly admitted correctional inmates. *American Journal of Public Health*.

[8] Wilper AP, Woolhandler S, Boyd JW (2009). The health and health care of US prisoners: results of a nationwide survey. *American Journal of Public Health*.

[9] Nobile CG, Flotta D, Nicotera G, Pileggi C, Angelillo IF (2011). Self-reported health status and access to health services in a sample of prisoners in Italy. *BMC Public Health*.

[10] Ware JE, Kosinski M, Dewey JE, Gandek B (2000). *SF-36 Health Survey Manual and Interpretation Guide*.

[11] Jenkinson C, Wright L, Coulter A (1994). Criterion validity and reliability of the SF-36 in a population sample. *Quality of Life Research*.

[12] Pappa E, Kontodimopoulos N, Niakas D (2005). Validating and norming of the Greek SF-36 Health Survey. *Quality of Life Research*.

[13] Anagnostopoulos F, Niakas D, Pappa E (2005). Construct validation of the Greek SF-36 Health Survey. *Quality of Life Research*.

[14] Pappa E, Kontodimopoulos N, Niakas D (2006). Psychometric evaluation and normative data for the Greek SF-36 health survey using a large urban population sample. *Archives of Hellenic Medicine*.

[15] Yfantopoulos J (2001). The Greek version of the EuroQol (EQ-5 D) instrument. *Archives of Hellenic Medicine*.

[16] Kontodimopoulos N, Pappa E, Niakas D, Yfantopoulos J, Dimitrakaki C, Tountas Y (2008). Validity of the EuroQoL (EQ-5D) instrument in a Greek general population. *Value in Health*.

[17] Dolan P (1997). Modeling valuations for EuroQol health states. *Medical Care*.

